# Homologous Targeting Effect of Cancer Cell-Derived Liposomes (Memposomes) Mediated by Cell Adhesion Molecules: Role of E-cadherin

**DOI:** 10.3390/biom14101212

**Published:** 2024-09-26

**Authors:** Hyein Cheung, Haewon Kang, Hyo Jung Lee, Yunjae Chung, Hanbo Shin, Sangmin Lee, Jong-Ho Kim

**Affiliations:** 1 College of Pharmacy and Bionanocomosite Research Center, Kyung Hee University, 26 Kyungheedae-ro, Dongdaemun-gu, Seoul 02447, Republic of Korea; 2 Department of Regulatory Science, Institute of Regulatory Innovation through Science, Graduated School, Kyung Hee University, 26 Kyungheedae-ro, Dongdaemun-gu, Seoul 02447, Republic of Koreahanbo0810@naver.com (H.S.)

**Keywords:** cell membrane-derived liposomes, Memposomes, drug delivery vehicle, homologous targeting, E-cadherin

## Abstract

Cell membrane-derived liposomes, termed Memposomes, serve as promising carriers for drug delivery due to their ability to closely mimic cells and efficiently target specific cells. Liposomes derived from cancer cell membranes, in particular, exhibit homologous targeting capabilities, making them potential candidates for cancer-specific drug delivery. However, the underlying mechanisms and specific proteins responsible for this homologous targeting phenomenon remain debated. This study focuses on the role of E-cadherin, a cell adhesion molecule implicated in homophilic adhesion, in influencing the homologous targeting ability of Memposomes derived from cancer cell membranes. E-cadherin expression patterns were assessed in various cell lines, categorizing them into E-cadherin-positive and -negative groups. Memposomes were produced for each group, and their targeting tendencies were evaluated. This study confirmed that E-cadherin expression significantly influenced the homologous targeting ability of the Memposomes. The cell lines with higher E-cadherin expression levels exhibited a more pronounced homologous targeting effect. This research demonstrates that cell adhesion molecules, particularly E-cadherin involved in homophilic adhesion, play a pivotal role in influencing the cell targeting ability of Memposomes. This study further validates the stability, safety, and purity of Memposomes, emphasizing their potential as effective drug delivery vehicles for the development of cell-specific therapies.

## 1. Introduction

Historically, cancer treatment strategies, encompassing surgical resection, chemotherapy, radiation therapy, and immunotherapy, have encountered considerable constraints, such as systemic toxicity, a lack of tumor specificity, and the emergence of drug resistance [1,2]. These limitations underscore the imperative to explore alternative strategies for cancer therapy.

Cancer therapy faces substantial challenges due to various characteristics of cancer cells, necessitating effective delivery systems [3]. The intricacies of cancer, including heterogeneity, aggressive growth, and resistance to treatment, emphasize the indispensability of developing precise and efficient therapeutic strategies [3,4]. In this context, drug delivery systems play a pivotal role in enhancing therapeutic outcomes by facilitating the targeted and controlled delivery of therapeutic agents to cancerous tissues while minimizing damage to healthy cells.

Targeted drug delivery systems, tailored to transport specific drugs with precision to the target tissues or cells, play a pivotal role in various medical applications, offering more effective cancer treatment compared to traditional chemotherapy [5,6]. The use of an appropriate drug delivery system holds the promise of achieving cancer cell targeting effects. A crucial element in successful cancer treatment is the specific targeting of cancer cells [7]. Moreover, drug delivery systems can maintain the stability of these drugs and safely transport them to the target tissues, ultimately leading to enhanced therapeutic effects and reduced side effects. In the absence of an appropriate delivery system, issues may arise, such as anticancer agents failing to precisely target the intended site, affecting surrounding normal cells, and causing severe side effects [8,9]. Additionally, anticancer drugs may degrade or be eliminated rapidly, limiting their therapeutic efficacy and necessitating frequent administrations, increasing patient discomfort [10]. Hence, the development and utilization of suitable drug delivery systems is critical in the quest for effective anticancer agents.

A promising approach involves the use of cancer cell-derived liposomes, exhibiting a homologous targeting effect [11]. The increasing interest in and utilization of cancer cell membrane-derived liposomes in recent years highlights their potential to revolutionize targeted drug delivery. Researchers have been exploring various cancer cell lines to produce of cancer cell membrane coating carriers that can more effectively target and treat specific types of cancer, thereby enhancing the precision and effectiveness of therapeutic interventions [12,13,14]. Furthermore, the concept of cancer cell membrane coatings has shown promise in evolving into more advanced forms which combine the natural targeting capabilities of cell membranes with the versatile drug-carrying capacity of liposomes. These liposomes, originating from cancer cell membranes, share surface characteristics with their parent cells, resulting in a higher affinity for interactions with the same or similar cancer cells [15,16]. This homologous targeting effect enhances the specificity and efficiency of drug delivery to cancerous tissues, potentially revolutionizing cancer therapy [17].

Although numerous studies have explored the homologous targeting effect of cancer cell-derived liposomes, the precise factors driving this phenomenon remain unclear [17,18,19,20,21]. Notably, cadherin family proteins and integrins have been implicated as significant contributors [22,23,24]. In particular, E-cadherin possibly plays an important role in homologous targeting because its homotypic adhesion, binding E-cadherin on the cancer cell surface and the E-cadherin on another cancer cell surface, facilitates selective binding and uptake [25,26]. In addition, E-cadherin-mediated adhesion can trigger endocytosis or other cellular internalization mechanisms [27,28,29]. Therefore, we hypothesized that E-cadherin, known for its role in homophilic adhesion within cell–cell adhesion molecules, could be a key protein in mediating the homologous targeting effect of liposomes.

To test this hypothesis, we selected various types of cancer cells and assessed the expression patterns of cadherin family proteins, including E-cadherin. Subsequently, liposomes were derived from these cancer cells and used to treat all selected cancer cells to evaluate their binding affinity. Our findings revealed that E-cadherin can induce a more pronounced homologous effect compared to other cadherins. In other words, cancer cells expressing higher levels of E-cadherin demonstrated a greater specificity in binding with liposomes derived from those same cells.

This study sheds light on the significant impact of cell–cell adhesion molecules, particularly E-cadherin, on the homologous targeting effect of liposomes. Furthermore, through various characterization experiments, we optimized the physicochemical properties of liposomes, reaffirming their potential as effective drug delivery carriers.

## 2. Materials and Methods

### 2.1. Materials and Cell Culture

HEPES buffer solution was purchased from Gibco Inc. (Waltham, MA, USA). Potassium chloride was purchased from JENSEI Inc. (Seongnam, Republic of Korea). Saccharose was purchased from DUKSAN Inc. (Ansan, Republic of Korea). EGTA buffer was purchased from Bio World Inc. (Dublin, OH, USA). The 0.5 M EDTA, pH 8.0 was purchased from USB Inc. (Dublin, OH, USA). Xpert Protease Inhibitor Cocktail Solution was purchased from genDEPOT Inc. (Baker, TX, USA). Fluorescein isothiocyanate (FITC) was purchased from Sigma Aldrich Inc. (St. Louis, MO, USA). Vybrant^tm^ DIO cell-labeling solution was purchased from Invitrogen Inc. (Carlsbad, CA, USA). Nuclepore^tm^ Track-Etch Membranes, 0.4 um and 0.1 um, were purchased from GE Healthcare Life Sciences Inc. (Seoul, Republic of Korea). Avanti Polar Lipid Extruder Kit and 10 mm Filter supports were purchased from Avanti Polar Lipids Inc. (Alabaster, AL, USA).

Rosewell Park Memorial Institute (RPMI) medium, Dulbecco’s modified eagle medium (DMEM) high glucose, and fetal bovine serum (FBS) were obtained from WelGENE Inc. (Daegu, Republic of Korea). Penicillin–streptomycin (PS) was purchased from Hyclone-GE Healthcare Bio-Science (Logan, UT, USA). H292, A549, MCF7, and MDAMB231 cells were purchased from American Type Culture Collection (ATCC, Manassas, VA, USA).

H292 andA549 were cultured in RPMI 1640 supplemented with 10% FBS and 1% PS. MCF7 and MDAMB231 were cultured in DMEM supplemented with 10% FBS and 1% PS. The cells were maintained in an incubation chamber with a humidified atmosphere at 37 °C and 5% CO_2_.

### 2.2. Characterization of Cells

Western blotting analysis was performed to investigate the composition of cell adhesion molecules. Briefly, the cell lysate was prepared using the RIPA lysis buffer, after which equal amounts of the proteins from different samples were quantified by BCA protein assay kit. All the samples with the sample buffer were heated to 90 °C for 10 min. Samples (30 ug/well) were loaded onto a 10% SDS-PAGE gel and run at 100 V for 2 h. The proteins on the gel were transferred to the PVDF membrane; then the membrane was blocked and incubated with the primary antibodies (1:1000) of anti-E-cadherin, N-cadherin, and b-actin at 4 °C overnight. The secondary antibodies (1:2000), horseradish peroxidase-conjugated anti-rabbit IgG or anti-mouse IgG, were incubated with corresponding primary antibodies at room temperature for 2 h before imaging with an ECL Western Blotting Substrate.

### 2.3. Preparation of Memposomes

**Figure 1:**
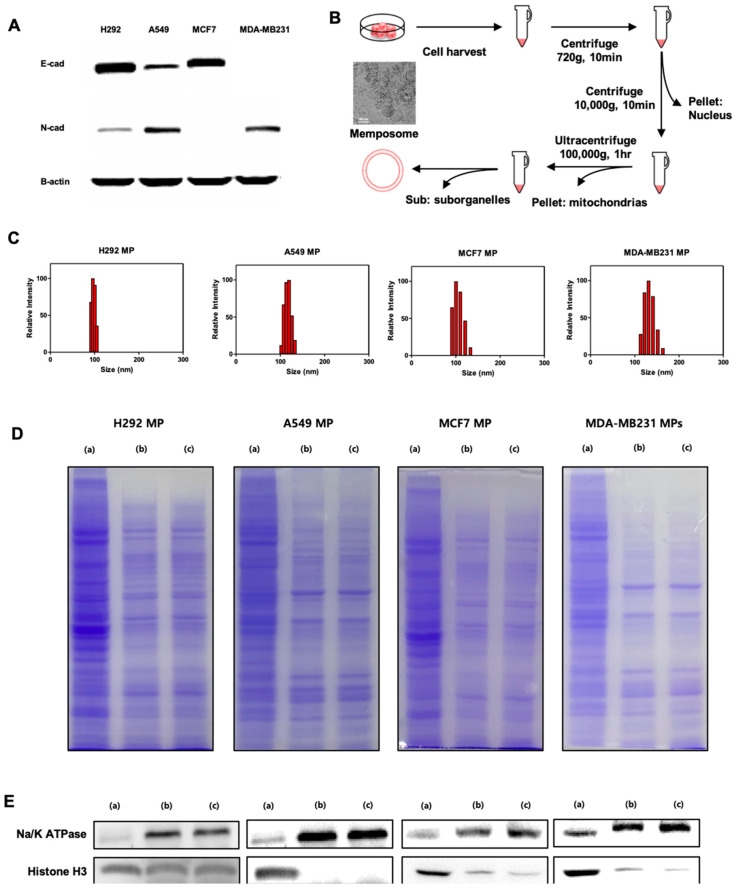
Preparation and characterization of MPs. (**A**) Western blot analysis of cell adhesion proteins. (**B**) Procedure of MP production. Insert image is TEM of MCF7 MPs. (**C**) Size of H292 MPs, A549 MPs, MCF7 MPs, and MDA-MB231 MPs measured by Dynamic Light Scattering. (**D**) SDS-PAGE protein analysis of cell lysates (**a**), cell membranes (**b**), and MPs (**c**). Samples were stained with Coomassie Blue. (**E**) Western blot analysis of cell-specific proteins (Na^+^/K^+^ ATPase: membrane marker, Histone H3: nucleosome) in cell lysates (**a**), cell membranes (**b**), and MPs (**c**) (from left to right: H292, A549, MCF7, and MDA-MB231). Original images of (**A**,**E**) can be found in Supplementary Materials.

The cell membrane was isolated from source cells (Figure 1B). Briefly, the cells were grown in 150 mm cell culture dishes to 90% confluency. To harvest the cell membrane, the cells were washed with phosphate-buffered saline (PBS) solution twice. Then, we collected the cells and added hypotonic buffer (250 nM sucrose, 20 nM HEPES pH = 7.4, 10 nM KCl, 1.5 nM MgCl_2_, 1 nM EDTA pH = 8.0, 1 nM EGTA, and 100 uL EDTA-free Protease Inhibitor Cocktail per 10 mL). The entire solution was sonicated for 20 min with a probe type sonicator (VCX 500, PRO Scientific Inc., Oxford, CT, USA) and centrifuged at 720× *g* for 5 min, after which we collected the supernatant. Then, the supernatant was centrifuged at 4 °C and 10,000× *g* for 10 min. After discarding the pellet, the supernatant was ultracentrifuged at 100,000× *g* for 1.5 h. The resulting pellet containing the plasma membrane material was resuspended with 2 M CaCl_2_ in DPBS. We incubated the mixture at 37 °C for 1 h. The mixture was centrifuged at 20,000× *g* for 10 min and washed with DPBS 3 times. The plasma membrane fraction was extruded through a polycarbonate membrane with pore sizes of 400 nm, 200 nm, and 100 nm to prepare cell membrane-derived liposomes, termed Memposomes (MPs).

### 2.4. Characterization of MPs 

The particle sizes of MPs were measured at a concentration of 100 ug/mL using a dynamic light scatter (DLS). H292 MPs, A549 MPs, MCF7 MPs, and MDAMB231 MPs were suspended in 3 mL DPBS and measurements were performed. The stability of MPs was measured using DLS. For storage stability, 300 ug of MPs was suspended in 3 mL in DPBS and stored at 4 °C, and the size was measured once a day. Serum stability determined by suspension in 5% FBS at the same concentration and with storage at 37 °C, and the size was measured 5 times a day.

For the sodium dodecyl sulfate polyacrylamide gel electrophoresis (SDS-PAGE) analysis, the cell lysate was prepared using the RIPA lysis buffer. All samples were heated to 90 °C for 10 min. Samples with equal protein amounts were loaded onto the 10% SDS-PAGE gel and run at 100 V for 2 h. Subsequently, the gel was stained with Coomassie blue for 2 h, followed by washing with destaining solution overnight. Pictures were taken with a camera for subsequent imaging.

For Western blotting assay, the proteins on the gel were transferred to PVDF membrane, and then the membrane was blocked and incubated with the primary antibodies of anti-Na/K ATPase, Histone H3, and b-actin at 4 °C overnight. The secondary antibodies, including either horseradish peroxidase-conjugated anti-rabbit IgG or anti-mouse IgG, were incubated with the corresponding primary antibodies at room temperature for 2 h before imaging with an ECL Western Blotting Substrate. The resulting gel was transferred to polyvinylidene difluoride membranes (PVDF) for Western blot analysis. Membranes were blocked with Histone H3 for nucleosome detection at 4 °C overnight, followed by incubation with horseradish peroxidase-labeled (HRP) goat anti-rabbit IgG cross-adsorbed secondary antibody (1:2000) for 2 h at 37 °C. The improved PICO solution was applied to the membrane and signals were detected by enhanced chemiluminescence (ECL) machine.

### 2.5. Cell Cytotoxicity

For in vitro cytotoxicity assay, the cells were seeded in 96-well plates at a density of 5 × 10^3^ cells per well and cultured for 18 h. Then, H292 MPs, A549 MPs, MCF7 MPs, and MDAMB231 MPs at various concentrations (i.e., 1, 2, 5, 10, 20, and 50 ug/mL) were added to the cells and the cells were incubated for 24 h. The cells without any particles were used as a control. Each group was repeated with six replicates. At the end of the incubation, 20 uL (5 mg/mL) Thiazolyl blue tetrazolium bromide (MTT) solution was added to each well, and the plate was incubated for 4 h. Finally, the solution was replaced by 150 uL of the DMSO solution. The absorbance values of the cells per well were determined with a microplate reader at 450 nm for analyzing the cell viability.

### 2.6. Targeting Tendency Binding MPs to Cells 

We conducted flow cytometry experiments to observe the cell-binding ability of MPs. MPs were first fluorescently labelled with fluorescein isothiocyanate (FITC) [30,31]. FITC-labeled MPs were characterized by measuring the absorbance at 494 nm by UV spectroscopy. An aliquot of 1 × 10^6^ live cells were dispersed in 200 uL of FACS buffer (2% BSA/PBS), after which 200 uL of FITC-labelled MPs (15 μg/mL) was added, and the mixture was incubated at 4 °C for 30 min. After washing with pre-cooled PBS, flow cytometry measurements were conducted; ten thousand events were collected for each measurement and analyzed by CytoExpert software version 2.6 (Beckman Coulter, Indianapolis, IN, USA). The mean intensity of blank H292, A549, MCF7, and MDAMB231 was chosen as control. The mean fluorescent intensity (MFI) was measured to quantify the amounts of MPs bound to cells and to compare the binding abilities of different MPs.

### 2.7. Staining MPs with DIO to Identify Targeting Tendency 

For fluorescence images, the cancer cell membrane was labelled by a cell membrane green fluorescent probe, DIO, as previously reported [32,33]. The cell membrane was suspended at a density of 500 μg/mL in DPBS and to this was added 5 μL of the DIO solution. The cell membrane solution with DIO was mixed well by gentle pipetting followed by incubation for 10 min at 37 °C. The DIO–cancer cell membrane was washed with PBS three times. For stabilization, the DIO–cancer cell membrane was incubated for 1 h and sonicated for 1 min, followed by extrusion for 15 cycles through 400 nm, 200 nm, and 100 nm polycarbonate porous membranes.

H292, A549, MCF7, and MDAMB231 cells were seeded in 24-well plates for fluorescence microscopy analysis. Then, the medium was replaced with the fresh medium contacting DIO-labelled MPs (15 ug/mL) and stained for 2 h. After washing three times with PBS, the cells were fixed and imaged with a fluorescence microscope.

### 2.8. Statistical Analysis 

In this study, the differences between control (same cell-derived MPs as were treated) and experimental groups were analyzed using one-way ANOVA and considered statistically significant if marked with an asterisk (* *p* < 0.05, ** *p* < 0.01) in the figures.

## 3. Results and Discussion

### 3.1. Characterization of Cells

We conducted Western blot experiments to assess the protein profile of the cells that would be used for MP production. In the Western blot experiment (Figure 1B), it was observed that H292 and MCF7 cells expressed significantly higher levels of E-cadherin compared to N-cadherin. On the other hand, A549 cells exhibited both E-cadherin and relatively higher expression of N-cadherin compared to other cell lines. Notably, MDA-MB231 cells lacked E-cadherin expression, which aligns with their known status as an E-cadherin negative control cell line. Based on the Western blot results, we classified H292 and MCF7 as having an abundance of E-cadherin, A549 as having a moderate level of E-cadherin, and MDA-MB231 as lacking E-cadherin. Subsequently, we proceeded with the experiments using these classifications.

### 3.2. Preparation and Characterization of MPs

To produce the MPs, we utilized the subcellular fractionation technique to isolate the plasma membrane fraction from the cell lysate. This method involves lysing the cells using a hypotonic buffer and then separating the cellular organelles through centrifugation. The obtained plasma membrane fraction contained membrane proteins. To convert this fraction into liposomes with a unilamellar structure, we subjected it to an extrusion process. Through the extrusion process, we were able to create uniform structures known as cell plasma membrane-derived liposomes or MPs. Using these cancer cell lines, we produced MPs and evaluated their physicochemical properties. To assess the particle size of the MPs, we measured the diameter of the MPs and observed that all four types of MPs had particles of approximately 100 nm in size (Figure 1C). The size and polydisperse index of the MPs are summarized in Table 1.

Additionally, to determine if proteins other than cell membrane proteins were effectively separated and if membrane proteins were well preserved, we conducted SDS-PAGE and Western blot analyses (Figure 1D,E). The results showed that the plasma membrane proteins were effectively separated from the cell lysate, and the MPs exhibited a similar protein composition to the plasma membrane fraction. This indicates that the MPs preserve the protein composition of the membrane fraction. Furthermore, we observed a significant reduction in the nuclear protein marker, Histone H3, in both the membrane fraction and the MPs compared to the whole cell lysate. However, the plasma membrane protein marker, Na/K ATPase, was detected in both the membrane fraction and the MPs. This result indicated that other cellular organelles apart from the plasma membrane were effectively removed, and there was no significant loss of the plasma membrane fraction. Therefore, it was confirmed that the MPs were well composed of cell plasma membrane and membrane-associated proteins.

### 3.3. Stability of MPs

**Figure 2:**
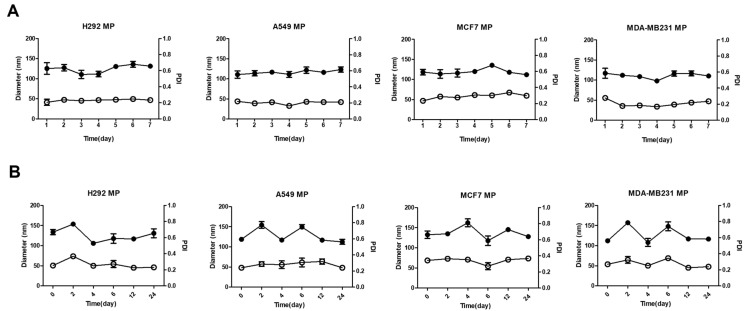
The stability of MPs. (**A**) Storage stability of H292 MPs, A549 MPs, MCF7 MPs, and MDA-MB231 MPs suspended in DPBS over time measured by DLS (4 °C) for 1 week. (**B**) Serum stability of H292 MPs, A549 MPs, MCF7 MPs, and MDA-MB231 MPs suspended in 5% FBS over time (37 °C) for 4 h (●: mean diameter, ○: polydisperse index (PDI)). Data are presented as means ± SDs (*n* = 5).

We conducted storage stability tests and serum stability tests to assess the stability of the MPs. In the storage stability test, the particle size of the MPs was measured at 24-h intervals for one week. Throughout the entire week, the polydispersity index (PDI) value remained consistently around 0.2 to 0.3, indicating good stability over time (Figure 2A). The researchers concluded that the particle size of the MPs remained stable over the course of the week, without any noticeable aggregation or significant changes. Additionally, the researchers tested the stability of the MPs in the presence of serum. They dispersed the MPs in phosphate-buffered saline (PBS) containing serum and measured the particle size at regular intervals over a 24-h period. The results showed that the particle size remained stable throughout the day, indicating that the MPs maintained their size stability even when exposed to serum (Figure 2B). However, compared to the storage stability test, the particle size and PDI value exhibited relatively less stability, suggesting some degree of variability. Nevertheless, the researchers noted that noticeable aggregation of the MPs did not occur, indicating that they maintained their overall size stability in the presence of serum over the course of 24 h. Overall, based on the experiments conducted, the researchers found that the MPs demonstrated good stability in terms of size and aggregation both during storage over a one-week period and when exposed to serum over a 24-h period.

### 3.4. Cytotoxicity of MPs

**Figure 3:**
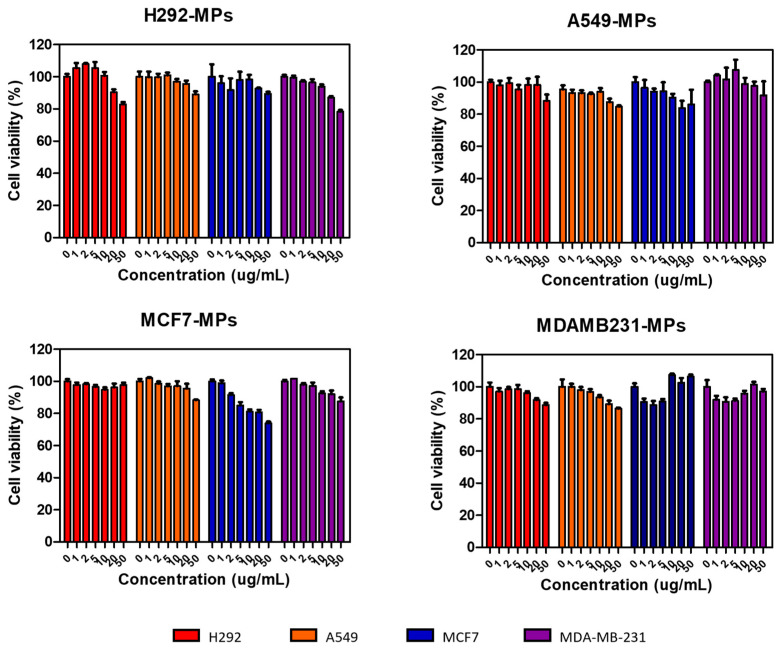
Cytotoxicity test of H292 MPs, A549 MPs, MCF7 MPs, and MDA-MB231 MPs in various cells after 24 h. H292, A549, MCF7, and MDAMB231 cells were incubated with different concentrations of MPs. Data are presented as means ± SDs (*n* = 5).

In the given study, we performed a cell viability assay to assess the cytotoxicity of different types of MPs that were derived from the cell membranes of specific cells. The purpose was to investigate their potential for cell binding while ensuring that they did not induce cytotoxicity. To conduct the assay, we treated each type of MP with a predetermined concentration and exposed them to four different cell lines. After a 24-h incubation period, we examined the cell viability to determine if the MPs had any detrimental effects on the cells (Figure 3). The results of the assay revealed that even at high concentrations of all types of MPs, the cell viability remained above 80%. This finding is significant as it indicates that the MPs derived from cancer cell plasma membranes do not induce cytotoxicity. Moreover, not only did they not show cytotoxic effects on the source cells of the MPs, they also did not on heterotypic cancer cell lines, suggesting their biocompatibility. This observation of high cell viability implies that the MPs possess favorable biocompatibility characteristics, making them suitable candidates for further investigation in biomedical applications. The lack of cytotoxic effects broadens their potential utility, as it suggests they could potentially be employed without causing harm to cells in various therapeutic or diagnostic contexts.

### 3.5. Targeting Ability of MPs

**Figure 4:**
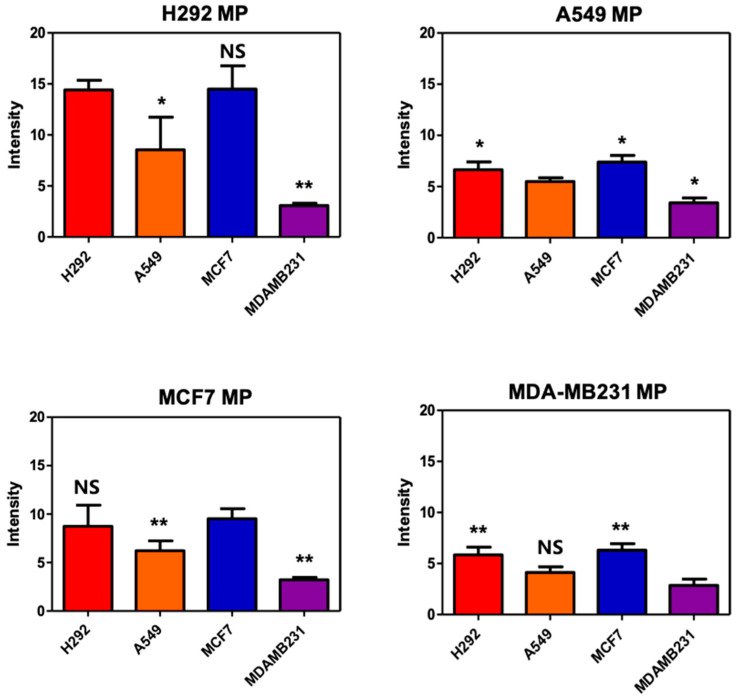
Quantification of flow cytometry analysis of H292 MPs, A549 MPs, MCF7 MPs, and MDAMB231 MPs in various cells. Data are presented as means ± SDs (*n* = 5). (*: *p* < 0.05, **: *p* < 0.01, NS: no significance).

In the provided text excerpt, we conducted a series of experiments to assess the composition, stability, and safety of MPs for potential utilization as drug delivery carriers. To delve further into the cell-binding characteristics of the synthesized MPs, the researchers conducted flow cytometry experiments with a specific emphasis on the quantification of E-cadherin, a cell adhesion molecule. In the flow cytometry experiment, the researchers labeled the MPs with FITC fluorescent dye, resulting in MP–FITC. Subsequently, they exposed four distinct cell lines to MP–FITC and subjected them to agitation at 4 °C for 30 min. Following this treatment, the measurement of bound MP–FITC on the cells allowed the determination of the extent to which the MPs had successfully adhered to the cells. The outcomes of this experiment unveiled a discernible correlation between the quantity of E-cadherin and the binding affinity of the MPs to the cells (Figure 4). Notably, the H292 cell line, characterized by the highest E-cadherin levels, exhibited a heightened propensity for binding the MPs compared to the other cell lines. This observation implies that cells expressing higher levels of E-cadherin possess an increased inclination to bind to the same type of MPs. Conversely, the MDA-MB231 cell line, lacking E-cadherin expression (E-cadherin negative), demonstrated a diminished affinity for binding the MPs relative to the other cell lines. This finding aligns with the established understanding that E-cadherin plays a pivotal role in facilitating cell–cell adhesion and binding interactions. Therefore, the researchers verified the composition, stability, and safety of the synthesized MPs, and the results from the flow cytometry experiments underscored the impact of E-cadherin on the cell-binding propensity of these particles. These insights contribute significantly to our comprehension of the factors influencing the interaction between MPs and cells, particularly in the context of drug delivery applications.

**Figure 5:**
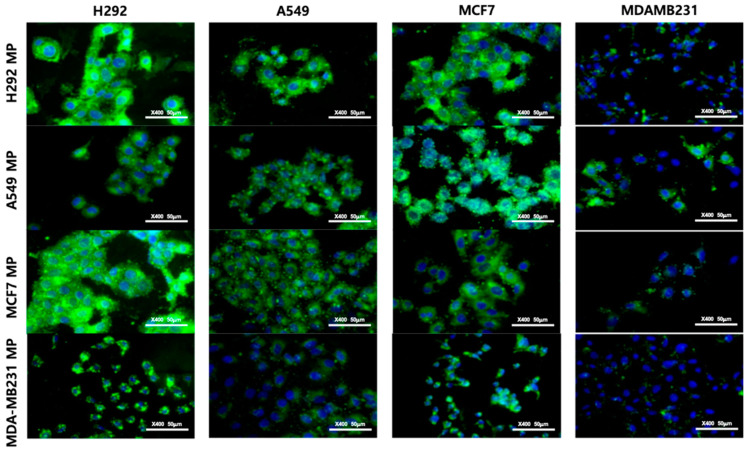
Fluorescence images of H292 MPs, A549 MPs, MCF7 MPs, and MDAMB231 MPs in various cells. Scale bar = 50 μm.

In addition to conducting the flow cytometry analysis, we employed fluorescent imaging to visually validate the obtained results. For this experiment, we employed a lipophilic fluorescent dye known as DIO (DIO–MP) to labelled MPs. Subsequently, the cells were treated with the labeled MPs, and fluorescent images were captured using the appropriate wavelength. The results obtained from the fluorescent imaging validated the trends observed in the flow cytometry data (Figure 5). Specifically, the H292 cell line demonstrated a robust binding affinity with nearly all types of MPs, surpassing the other cell lines in terms of binding strength. In contrast, the MDA-MB231 cell line exhibited considerably lower levels of binding with the MPs compared to the rest of the cell lines. The A549 and MCF7 cell lines displayed intermediate levels of MP binding. Thus, by integrating fluorescent imaging with flow cytometry, we were able to visually confirm the binding characteristics of the labeled MPs and gain further insights into the differential binding patterns exhibited by various cell lines.

The findings of this study highlight the potential of MPs, or cell membrane-derived liposomes, as a promising platform for targeted drug delivery. The ability of the MPs to mimic the surface characteristics of their source cells facilitates homologous targeting, which is particularly advantageous for cancer therapy. A key discovery from this research is the significant role E-cadherin plays in enhancing the homologous targeting ability of the MPs. E-cadherin, a cell adhesion molecule involved in homophilic adhesion, was identified as a crucial factor that influences the binding affinity of the MPs to their target cells. The MPs derived from E-cadherin-positive cells demonstrated a higher targeting efficiency towards cells with similar E-cadherin expression levels. This finding supports the hypothesis that cell adhesion molecules such as E-cadherin are pivotal in mediating the specificity of MPs in targeting homologous cells. These results carry significant implications for the development of targeted cancer therapies. By harnessing the homologous targeting capabilities of MPs, it may be possible to design drug delivery systems that selectively target cancer cells while minimizing damage to healthy tissues. This specificity could enhance the efficacy of anticancer treatments and reduce the side effects associated with conventional therapies.

## 4. Conclusions

Through the conducted experiments, it became evident that the homologous targeting effect of MPs, which are liposome-like structures created from cell membranes, varies based on the characteristics of the source cell membrane. Notably, when the MPs were derived from cancer cell membranes characterized by high E-cadherin expression, they exhibited a remarkable homologous targeting effect. Conversely, the MPs originating from cancer cell membranes lacking E-cadherin displayed a less pronounced homologous targeting effect. This observation implies that MPs effectively mimic the functionality of cancer cell membranes. By considering the protein profile expressed on cell membranes, we have the capability to design MPs with diverse functionalities. The roles of membrane proteins vary depending on the cell type, and a comprehensive understanding of these roles could pave the way for targeted therapies utilizing MPs. This research underscores the utility of MPs as effective drug delivery vehicles and establishes their physicochemical stability. We confirmed that cell membrane proteins are well-preserved in MPs, and effective separation is achieved from proteins originating from other cellular organelles. Furthermore, the stable maintenance of the particle size for over a week was observed. Consequently, MPs exhibit both safety and stability as drug delivery vehicles. These findings contribute to the growing body of evidence supporting the potential of MPs for targeted drug delivery applications. For future studies, it would be beneficial to explore the targeting efficiency of MPs in vivo and investigate the potential roles of other cell adhesion molecules in homologous targeting. Additionally, evaluating the therapeutic efficacy of MP-encapsulated drugs in animal models would provide valuable insights into their clinical potential.

## Figures and Tables

**Table 1:** Characterization of MPs. Size and PDI were measured by dynamic light scattering.

MP	Size (nm) ^1^	PDI ^2^
H292	98.3 ± 6.0	0.206
A549	116.6 ± 14.5	0.219
MCF7	110.3 ± 18.9	0.234
MDA-MB231	136.3 ± 22.8	0.277

^1^ diameter (mean ± S.D). ^2^ polydisperse index.

## Data Availability

The original contributions presented in the study are included in the article/Supplementary Material, further inquiries can be directed to the corresponding authors.

## Supplementary Materials

The following supporting information can be downloaded at: https://www.mdpi.com/article/10.3390/biom14101212/s1, Original images of Figure 1A,E can be found in Supplementary Materials.
