# Supplementary material for: Homologous Targeting Effect of Cancer Cell-Derived Liposomes (Memposomes) Mediated by Cell Adhesion Molecules: Role of E-cadherin
Source: Biomolecules. 2024 Sep 26;14(10):1212. doi: 10.3390/biom14101212 (PMC11506462; doi:10.3390/biom14101212)
Supplement: Supplementary file 1 [file biomolecules-14-01212-s001.zip › biomolecules-3150819-supplementary.pdf]

Figure 1A

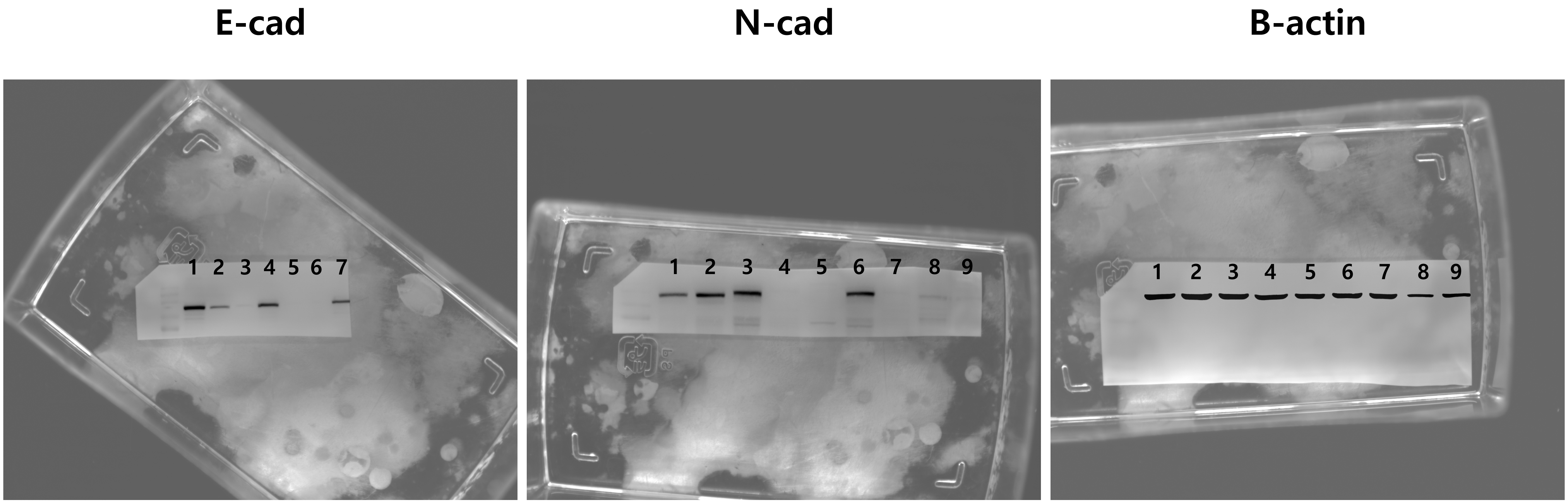

Lane 1: H292
Lane 2: A549
Lane 3: H1299
Lane 4: MCF7
Lane 5: T27D
Lane 6: MDA-MB231
Lane 7: AsPC1
Lane 8:HT29
Lane 9: HEK293

Figure 1E: H292

Na/K ATPase

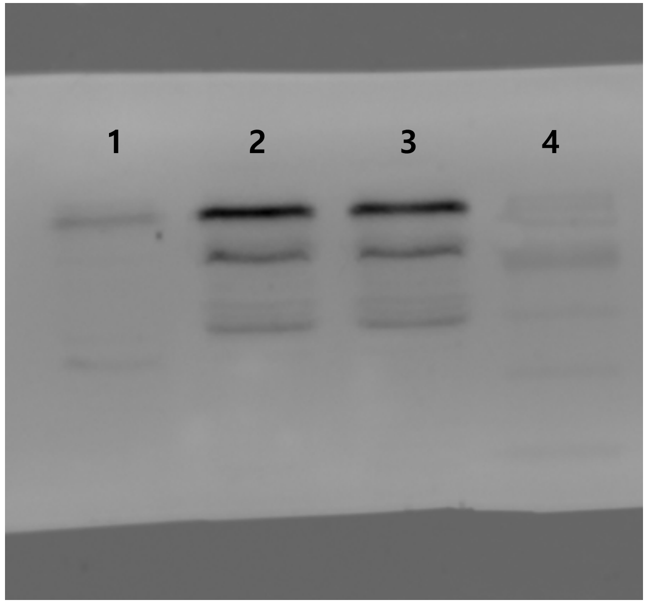

Lane 1: cell lysate
Lane 2: cell membrane
Lane 3: MP
Lane 4: Marker

Histone H3

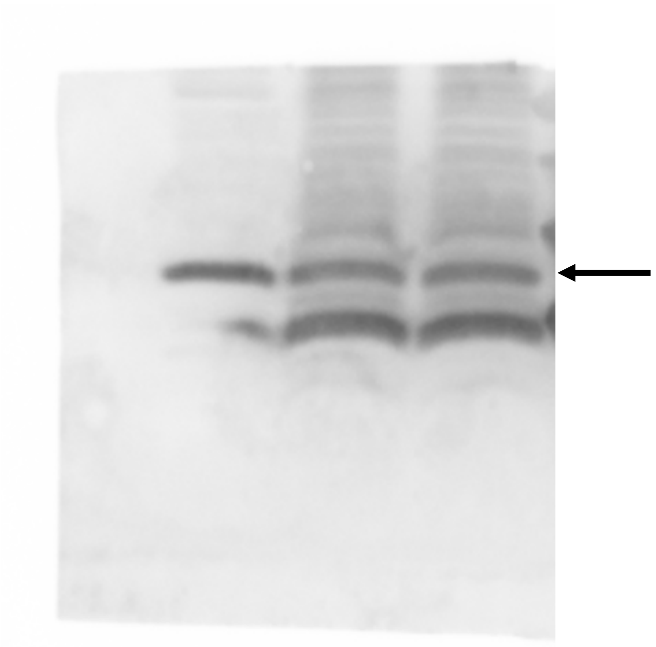

Lane 1: cell lysate
Lane 2: cell membrane
Lane 3: MP

Figure 1E: A549

Na/K ATPase

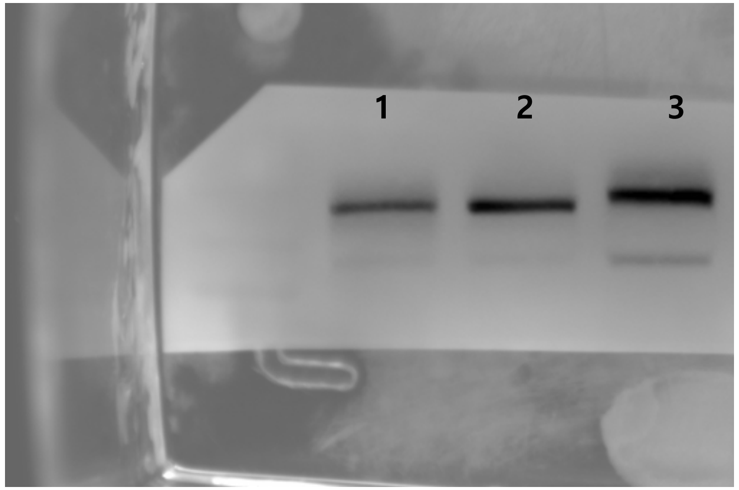

Lane 1: cell lysate
Lane 2: cell membrane
Lane 3: MP

Histone H3

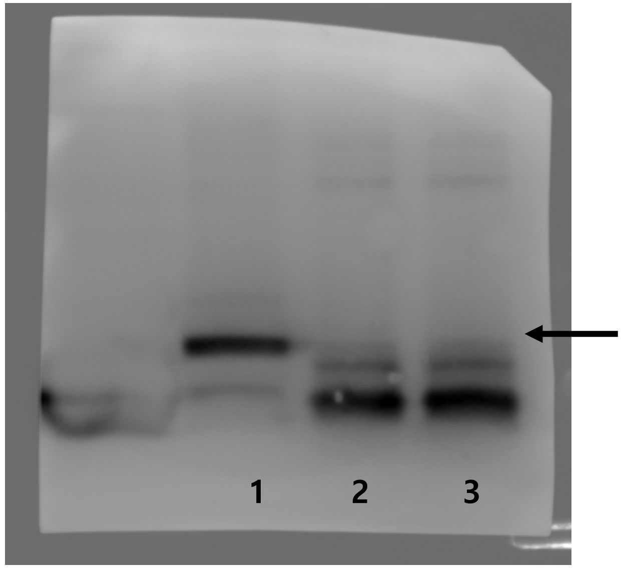

Lane 1: cell lysate
Lane 2: cell membrane
Lane 3: MP

Figure 1E: MCF7

Na/K ATPase

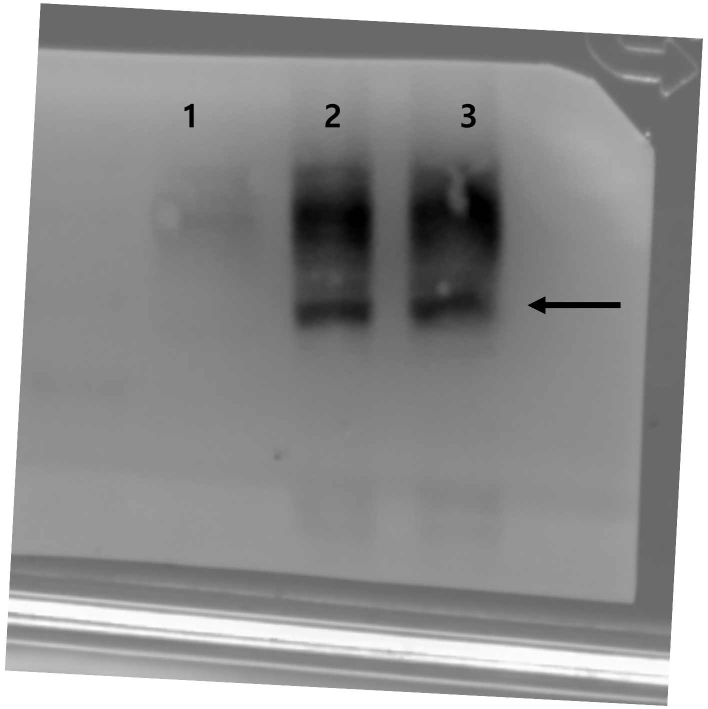

Lane 1: cell lysate
Lane 2: cell membrane
Lane 3: MP

Histone H3

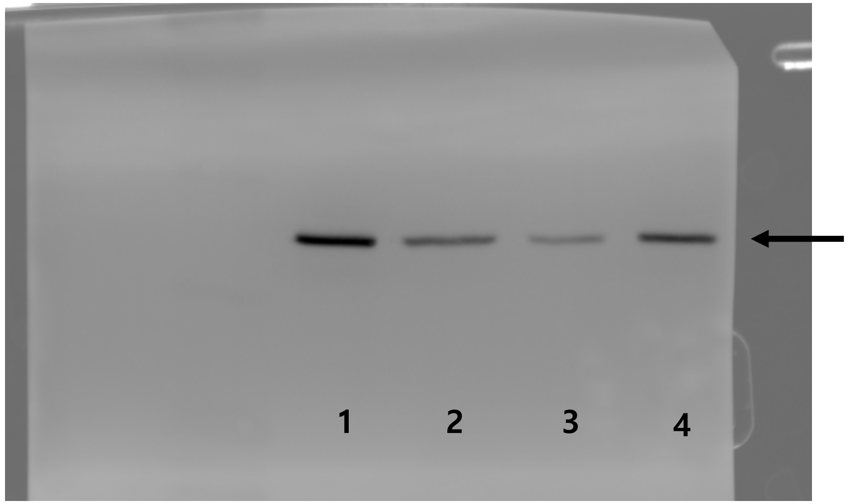

Lane 1: cell lysate
Lane 2: cell membrane
Lane 3: MP
Lane 4: another

Figure 1E: MDA-MB231

Na/K ATPase

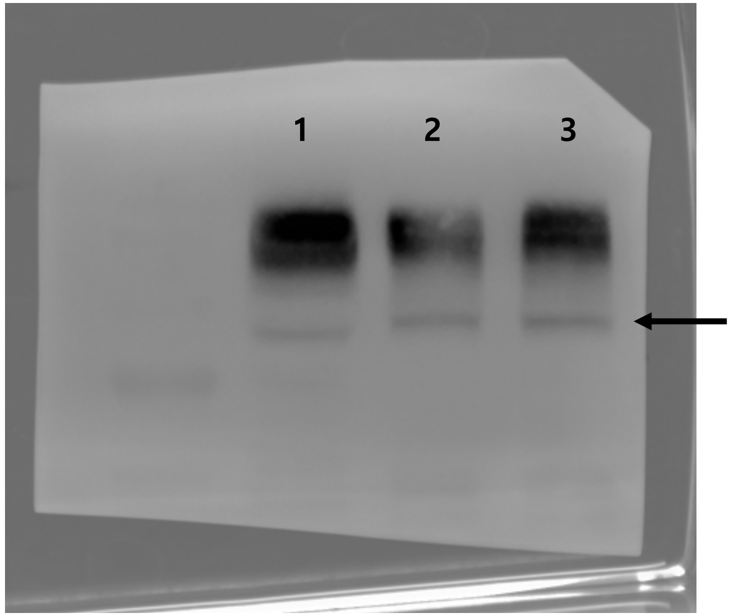

Lane 1: cell lysate
Lane 2: cell membrane
Lane 3: MP

Histone H3

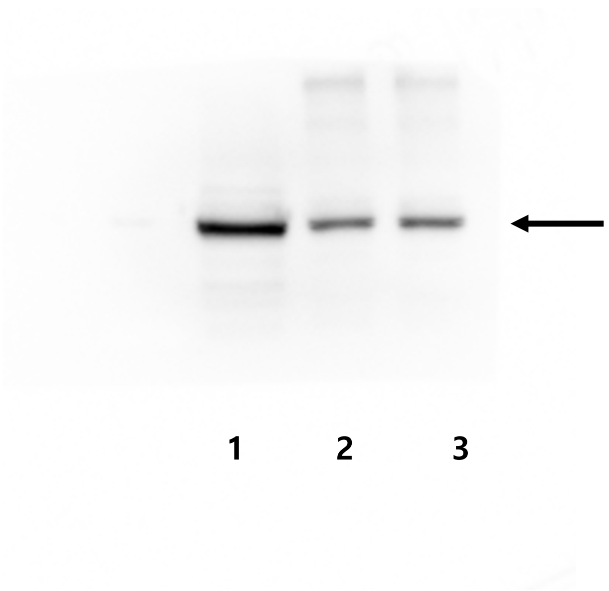

Lane 1: cell lysate
Lane 2: cell membrane
Lane 3: MP
